# Altered DNA methylation in estrogen-responsive repetitive sequences of spermatozoa of infertile men with shortened anogenital distance

**DOI:** 10.1186/s13148-022-01409-1

**Published:** 2022-12-26

**Authors:** Ludwig Stenz, Matthias Beyens, Mark E. Gill, Ariane Paoloni-Giacobino, Christian De Geyter

**Affiliations:** 1 Department of Genetic Medicine and Development, Faculty of Medicine, University of Geneva, Rue Michel-Servet, 1, 1211 Geneva, Switzerland; 2 Swiss Centre for Applied Human Toxicology (SCAHT), Missionsstrasse, 64, 4055 Basel, Switzerland; 3 Reproductive Medicine and Gynecological Endocrinology (RME), University Hospital, University of Basel, Vogesenstrasse, 134, 4031 Basel, Switzerland; 4 BISC Global, Bioinformatics and Statistics Consulting, Gaston Crommenlaan, 8, 9050 Ghent, Belgium

**Keywords:** Infertility, Protamine, Apoptosis, Methylation, Estrogen response element, Spermatozoa, Anogenital distance, Epigenome, Histone, Testicular dysgenesis syndrome

## Abstract

**Background:**

It has been suggested that antenatal exposure to environmental endocrine disruptors is responsible for adverse trends in male reproductive health, including male infertility, impaired semen quality, cryptorchidism and testicular cancer, a condition known as testicular dysgenesis syndrome. Anogenital distance (AGD) is an anthropomorphic measure of antenatal exposure to endocrine disruptors, with higher exposure levels leading to shortened AGD. We hypothesized that exposure to endocrine disruptors could lead to changes in DNA methylation during early embryonic development, which could then persist in the sperm of infertile men with shortened AGD.

**Results:**

Using fluorescence activated cell sorting based on staining with either YO-PRO-1 (YOPRO) or chromomycin-3 (CMA3), we isolated four sperm fractions from eleven infertile men with short AGD and ten healthy semen donors. We examined DNA methylation in these sorted spermatozoa using reduced representation bisulfite sequencing. We found that fractions of spermatozoa from infertile men stained with CMA3 or YOPRO were more likely to contain transposable elements harboring an estrogen receptor response element (ERE). Abnormal sperm (as judged by high CMA3 or YOPRO staining) from infertile men shows substantial hypomethylation in estrogenic Alu sequences. Conversely, normal sperm fractions (as judged by low CMA3 or YO-PRO-1 staining) of either healthy donors or infertile patients were more likely to contain hypermethylated Alu sequences with ERE.

**Conclusions:**

Shortened AGD, as related to previous exposure to endocrine disruptors, and male infertility are accompanied by increased presence of hormonal response elements in the differentially methylated regulatory sequences of the genome of sperm fractions characterized by chromatin decondensation and apoptosis.

**Supplementary Information:**

The online version contains supplementary material available at 10.1186/s13148-022-01409-1.

## Introduction

In numerous countries worldwide, the total fertility rate (TFR), which is defined as the average number of children born to a woman during her lifetime, has been in decline for decades [1]. In most industrialized countries, TFR has dropped well below the threshold level needed to sustain current population levels [2]. Both in Western [3–5] and Eastern [6] industrialized countries, this decline is accompanied by decreases in sperm count and lower age-corrected circulating testosterone levels [7–9]. In those same geographic areas, the incidence of both testicular cancer [10] and cryptorchidism [11], each strongly associated with reduced male fertility [12–14], are on the rise. These observations jointly suggest that impaired male fertility is at least one of several influential factors involved in the decline of TFR in industrialized countries. The evidence for an environmental origin of these associations results from comparisons of concurrent incidence trends occurring simultaneously [10]. Shifting incidences of testicular cancer in second-generation migrants from countries with low to high incidence countries [15, 16] or vice versa [17] also point toward an environmental origin. Low semen count, testicular cancer and cryptorchidism have all been associated with ante- and perinatal exposure to endocrine disrupting compounds [10, 14, 18]. The exact mechanisms, through which the disruptive changes of environmental pollutants on gonadal development are exerted, remain unknown.

In the human embryo, primordial germ cells arise in the epiblast of post-implantation embryos approximately 9 to 11 days after fertilization and primordial germ cells are formed in the yolk sac until approximately week 5 of early embryonic growth [19, 20]. During that early stage of embryonic development, the nuclear chromatin of the germ cells is completely reorganized through global DNA demethylation. During later gametogenesis, global re-methylation of the genome occurs when male and female germ cell development diverges to produce either spermatozoa or oocytes, resulting in globally higher DNA methylation in sperm and a re-setting of genomic imprints. The crucial importance of this tightly regulated process for later fertility is evidenced by the targeted disruption of DNMT3L, one of the key factors involved in methylation of CpG dinucleotides, which results in complete azoospermia in homozygous mutant male mice [21]. However, demethylation during early gametogenesis also opens a window for more subtle changes in the transcriptional regulation of genes [22–25]. Open chromatin in TEs of early primordial germ cells may allow usage of binding sites for regulatory elements of gene function, including transcription factors [26–28]. Alu repeat sequences in particular have been associated with hormone response elements [29, 30], which may be a target for endocrine disrupting compounds.

After formation of the primordial germ cells, male specific re-methylation resumes, and during ongoing gametogenesis, germ cells undergo rapid proliferation. Newly formed germ cells with disrupted gene regulation are prone to be eliminated at an early stage of gametogenesis through apoptosis, whereas others with more favorable characteristics may thrive ultimately leading to the formation of subpopulations of spermatogonia with slight variances in their gene regulatory patterns. As a result, cohorts of mature spermatozoa with differences in histone content and chromatin density emerge [31, 32]. Spermatozoa with aberrant chromatin condensation left with more histones in their nuclei are more susceptible to damage exerted by exposure to oxygen radicals resulting in higher numbers of spermatozoa with fragmented DNA [33–36].

Endocrine disruptors may act through either genomic or non-genomic estrogenic signaling pathways [37, 38]. They exert their function in the genome either directly through binding to estrogen response elements (ERE) in the regulatory elements of target genes or through interference with co-regulating transcription factors, which may either act on ERE or on alternative regulatory elements in estrogen target genes. The anogenital distance (AGD) describes the distance from the anus to the scrotum and reflects exposure to endocrine disruptors during prenatal development [39, 40], whereas the relationship between shortened AGD and reduced sperm count remains somewhat controversial [41–43], both testicular cancer and cryptorchidism occur more frequently in individuals with shortened AGD [44–47].

We here hypothesize that infertile men with shortened AGD produce distinct subpopulations of spermatozoa with differentially methylated genomic regions and that a significant proportion of these differentially methylated genomic regions carry ERE, through which endocrine disruptors may have mediated their signaling effects antenatally. These DNA methylation changes could result in subtle differences in sperm function, leading to changes in regulation of genes potentially contributing to the reduced fertility of those men, even in the presence of seemingly normal semen quality, as given by conventional semen analysis.

## Results

### Characteristics of participants to the experimental study

Eleven men presenting with infertility and ten young healthy men, all approved donors of semen for insemination, were asked to provide a semen sample for the sorting of spermatozoa in the frame of this experimental study. The overall characteristics of the participants are given in Table 1. The mean AGD of the infertile patients was significantly shorter than the mean AGD of the healthy semen donors (*p* < 0.0001) and the DNA fragmentation rate of the spermatozoa of the infertile patients, as measured by TUNEL, exceeded 20% in all cases, corresponding to the selection criteria of the study protocol. The healthy donors were significantly younger than the infertile patients (*p* < 0.01) and presented with larger testicular volumes (*p* < 0.01). All participating men had essentially normal semen counts, which was essential in order to have enough spermatozoa for FACS. Of note, although the difference did not reach statistical significance (*p* = 0.066), the circulating levels of FSH of the infertile patients with shortened AGD were slightly higher as compared to those of the healthy semen donors, suggesting subtle differences in Sertoli cell function.

**Table 1:** Descriptive statistics of the characteristics of the participating individuals

Parameter	Infertile patients (*n* = 11)				Healthy semen donors (*n* = 10)				p value
	Median	Mean	SD	Range	Median	Mean	SD	Range	
Age (y.)	42.0	41.5	4.9	35–50	33.0	34.9	5.8	27–44	0.006
BMI (kg/m^2^)	24.9	24.8	3.3	21.7–31.4	23.3	23.5	2.9	20.0–29.3	0.123
RR systolic (mm Hg)	128.0	129.5	15.6	109–155	124	127.4	14.8	111–157	0.429
RR diastolic (mm Hg)	74.0	75.1	9.6	63–93	75.5	73.5	10.2	55–91	0.500
AGD (mm)	35.0	35.0	7.4	25–44	69.0	70.2	8.2	58–87	< 0.0001
Testicular volume (ml)	42.0	33.9	11.8	22–55	69.0	50.0	51.0	40–70	0.004
LH (IU/l)	4.5	4.7	1.4	3.0–7.7	5.1	4.8	1.3	2.5–6.5	0.363
FSH (U/l)	5.2	4.9	1.8	1.8–8.0	2.7	3.7	2.1	1.7–8.1	0.066
Testosterone (nmol/l)	14.9	18.1	9.2	7.0–33.6	21.2	20.5	5.4	12.6–30.6	0.138
Sperm concentration (mill./ml)	43.7	69.4	48.9	19.3–158	57.1	71.3	55.7	18.1–211	0.484
Progressive motility (%)	44.0	42.2	20.2	14–80	44.5	47.0	14.0	28–72	0.298
Normal morphology (%)	13.6	12.5	5.8	1.8–21.6	10.5	11.3	4.3	5.9–18	0.323
DNA fragmentation (%)	32.5	29.5	8.6	22.1–51.5					

### Normalized CpG methylation levels of sorted spermatozoa of infertile patients as compared to those of sorted spermatozoa of healthy semen donors

**Fig. 1:**
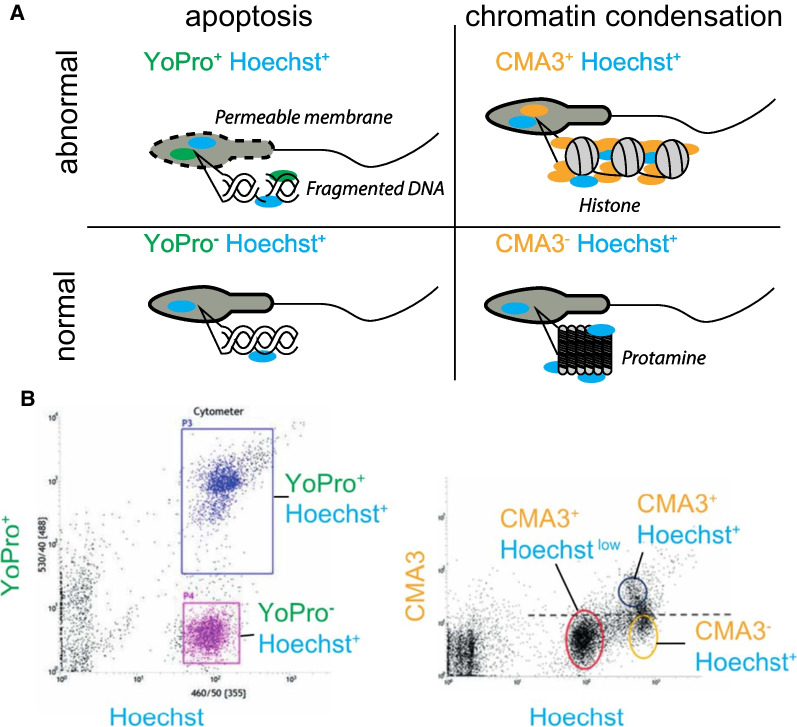
Sorting of spermatozoa with FACS. Four fractions of spermatozoa of infertile patients and healthy semen donors were separated with the use of FACS. **A** The YOPRO1-dye was used for separating apoptotic spermatozoa based on their membrane permeability and has previously been shown to correlate well with the degree of fragmentation of sperm DNA [54]. The chromomycin 3-dye (CMA3) specifically binds to guanosine cytosine-rich sequences in the DNA that compete with protamine and high CMA3− uptake correlates with reduced chromatin condensation and high histone content, whereas low CMA3− uptake correlates with high chromatin condensation [55, 56, 58]. **B** Representative examples of sorting results for the staining with YOPRO1 and CMA3. The Hoechst 33342-dye or Vybrant Dye Cycle Ruby was used to identify and remove debris from the samples

We used FACS to sort cohorts of spermatozoa based on DNA fragmentation, as given by YO-PRO-1 (YOPRO), and enhanced histone retainment and protamine deficiency, as given by chromomycin-3 (CMA3) (Fig. 1).

**Fig. 2:**
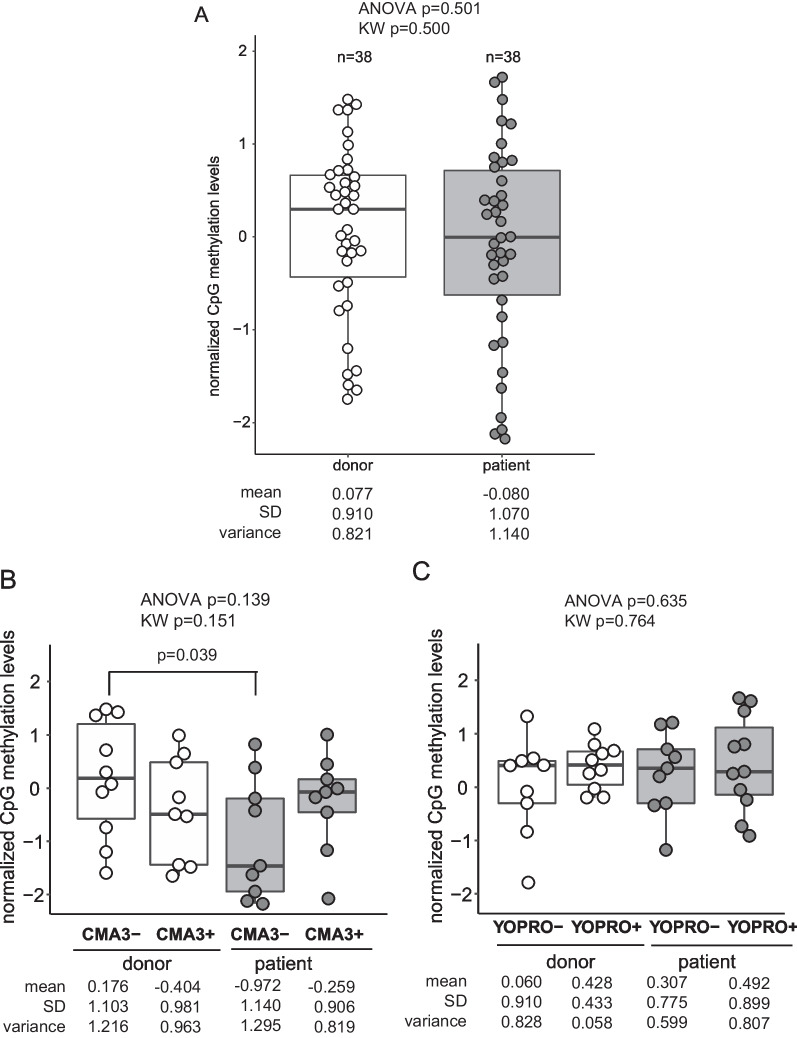
Global characterization of methylation status of the sperm populations. Normalized CpG methylation levels were compared in the pooled sperm fractions of healthy semen donors and infertile patients with shortened AGD (**A**), in the sperm fractions of both groups of participants based on uptake or non-uptake of CMA3 (**B**) and based on uptake or non-uptake of YOPRO (**C**). Normalized CpG methylation levels significantly lower in CMA3− negative sperm fraction (CMA3−) of infertile patients as compared to that of healthy donors (*p* = 0.039). No differences in normalized CpG methylation levels were detected in the sperm fractions sorted based on YOPRO− uptake. Differences in the normalized CpG levels with examined both with ANOVA and with Kruskal–Wallis

As summarized in Additional file 1: Table S1, we compared the differentially methylated CpG’s in the nuclear chromatin of 38 samples of sorted spermatozoa recovered from healthy donors with 38 matched samples of sorted spermatozoa of infertile patients (Fig. 2A). The normalized CpG methylation levels did not reveal any statistically significant difference among both groups (analyzed both with ANOVA and Kruskal–Wallis). Overall, the variance of the normalized CpG methylation levels was considerable, but more pronounced in the samples provided by the infertile patients (1.140 vs. 0.821).

We next compared the normalized CpG methylation levels in the fractions of sorted spermatozoa of either healthy semen donors or infertile patients grouped based on the uptake of either CMA3 (Fig. 2B) or YOPRO (Fig. 2C). Normalized CpG methylation levels were significantly lower in CMA3− negative sperm fraction (CMA3−) of infertile patients as compared to those of healthy donors (*p* = 0.039). The distribution of CpG methylation levels based on uptake of YOPRO did not reveal any differences in the sorted sperm fractions of both healthy donors and infertile patients.

### Comparison of the number of estrogenic and non-estrogenic short transposable sequences in the DMR of sorted spermatozoa stained with or without CMA3

Through the process of retro-transposition, Alu sequences together with other short TEs may become integrated into the demethylated genome of early primordial germ cells. Alu sequences frequently contain hormone response elements [29, 30], most particularly ERE [48]. Among 1′058′101 genomic Alu sequences identified in the current data sets, 180′769 contained ERE (17.1%). Estrogen receptors, activated by endocrine disruptors, may then bind to ERE in the short TEs, most particularly Alu sequences, and in conjunction with DNA (cytosine-5)-methyltransferase (DNMT) or other regulatory factors interfere with the global re-methylation during continued gametogenesis.

**Fig. 3:**
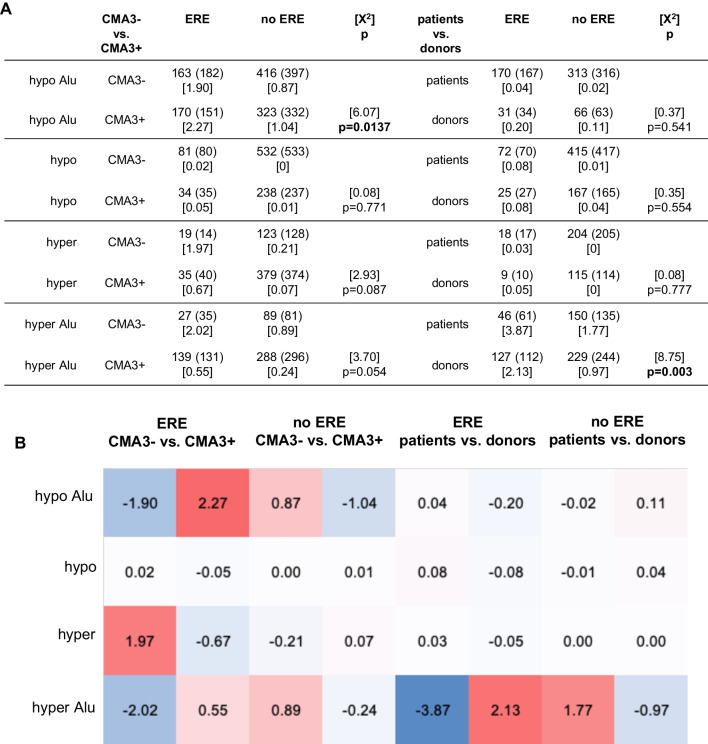
ERE in the sperm fractions sorted based on CMA3− uptake. We compared the observed number of differentially methylated estrogenic and non-estrogenic Alu sequences and other short transposable sequences based on the expected number in the fractions of spermatozoa either stained or not with CMA3 and in the sperm fractions of infertile patients versus healthy semen donors. Differences between the observed and expected numbers were evaluated with the Chi-squared (X^2^)-test (degrees of freedom, DoF: 1) (**A**). The observed number of hypomethylated estrogenic Alu sequences in the condensed CMA3− sperm fraction of infertile patients was significantly lower than expected, whereas the observed number of hypomethylated estrogenic Alu sequences was significantly higher in the CMA3+ sperm fraction of infertile patients (*p* = 0.0137). Conversely, the observed number of hypermethylated estrogenic Alu sequences was significantly higher than the expected number in the CMA3− fraction of fertile semen donors (*p* = 0.003). Based on the calculated X^2^-values of the estrogenic sequences, heat maps were constructed to visualize differences between observed and expected number of sequences based on CMA3− uptake (**B**). In the comparisons, in which the observed numbers exceeded the expected number, the X^2^-values were colored red (positive), whereas in the comparisons, in which the observed number remained below the expected number, the X^2^-values were colored blue (negative)

Due to suspected antenatal exposure of the infertile patients with shortened AGD, we hypothesized differences in the number of differentially methylated estrogenic Alu and other short TEs in the fractions of spermatozoa of infertile patients. In correspondence to the initial hypothesis, the observed number of hypomethylated estrogenic Alu sequences in the CMA3− sperm fraction of infertile patients was significantly lower than expected, whereas the observed number of hypomethylated estrogenic Alu sequences was significantly higher in the CMA3+ sperm fraction of infertile patients (*p* = 0.0137, Fig. 3). Conversely, the observed number of hypermethylated estrogenic Alu sequences was significantly higher than the expected number in the CMA3− fraction of the fertile semen donors (*p* = 0.003, Fig. 3).

### Comparison of the number of estrogenic and non-estrogenic short transposable sequences in the DMR of sorted spermatozoa stained with or without YOPRO1

**Fig. 4:**
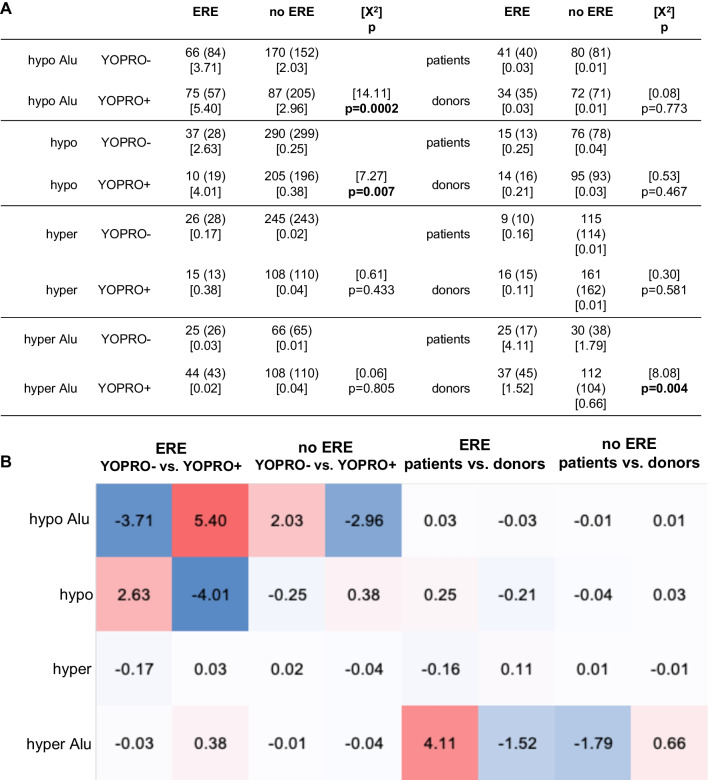
ERE in the sperm fractions sorted based on YOPRO− uptake. We compared the observed number of differentially methylated estrogenic and non-estrogenic Alu and other short transposable sequences based on the expected number in the fractions of spermatozoa either stained or not with YOPRO and in the sperm fractions of infertile patients versus healthy semen donors (**A**). The observed number of hypomethylated estrogenic Alu sequences and of other short transposable sequences significantly differed from the expected numbers in YOPRO− and YOPRO+ sperm fractions of infertile patients (*p* = 0.0002, resp. *p* = 0.007). The fraction of spermatozoa remaining unstained with YOPRO in infertile patients was more likely to contain hypermethylated estrogenic Alu sequences, whereas the fraction of spermatozoa stained with YOPRO was more likely to contain hypermethylated estrogenic Alu sequences (*p* = 0.004). Based on the X^2^-values of the estrogenic and non-estrogenic sequences, heat maps were constructed to visualize differences between observed and expected number of sequences based on YOPRO− uptake (**B**). In the comparisons, in which the observed numbers exceeded the expected number, the X^2^-values were colored red (positive), whereas in the comparisons, in which the observed number remained below the expected number, the X^2^-values were colored blue (negative)

We then compared the number of differentially methylated Alu sequences and other TEs containing or not containing ERE in the fractions of spermatozoa sorted based on YO-PRO-1-uptake in either infertile patients or healthy semen donors (Fig. 4). The observed number of hypomethylated estrogenic Alu sequences and of other short TEs significantly differed from the expected numbers in YOPRO− and YOPRO+ sperm fractions of infertile patients (*p* = 0.0002, resp. *p* = 0.007). In addition, the fraction of spermatozoa remaining unstained with YOPRO in infertile patients was more likely to contain hypermethylated estrogenic Alu sequences (*p* = 0.004).

### Genes associated to estrogenic or to non-estrogenic DMR

**Fig. 5:**
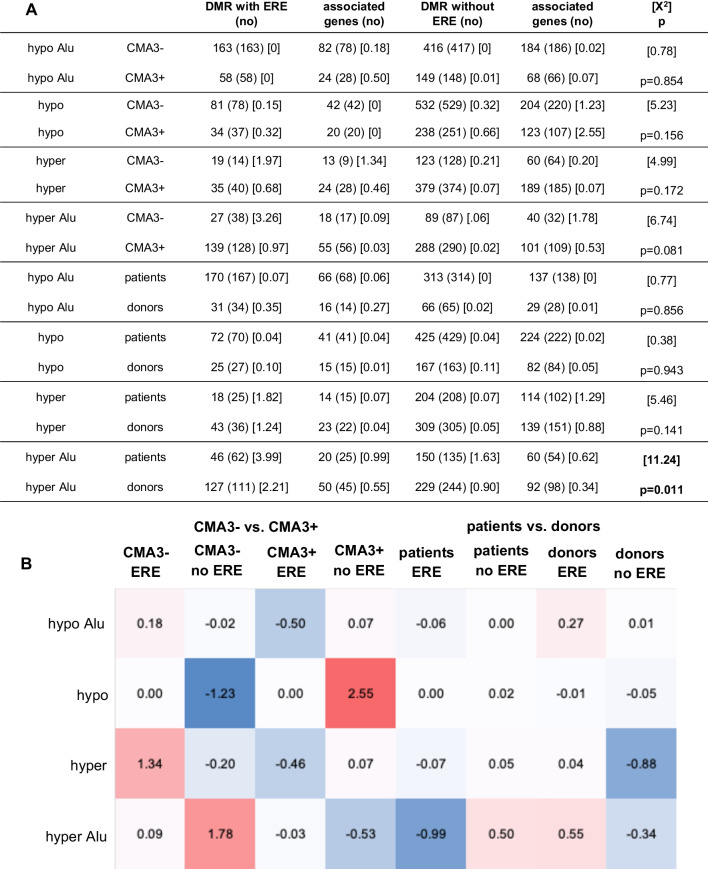
Genes associated to estrogenic or non-estrogenic transposable regulatory sequences in sperm fraction sorted based on CMA3− uptake. The observed number of genes associated to the DMR in the sorted sperm fractions of infertile patients and healthy semen donors and their relationship with ERE in the regulatory segments of those genes were compared with the expected numbers and differences between both were evaluated using chi-squared (X^2^)-tests (**A**). The number of genes associated with differentially methylated regulatory sequences containing or not containing ERE was similar in the CMA3− sorted sperm fractions of infertile patients and healthy donors. Based on the X^2^-values, heat maps were constructed to visualize differences between observed and expected numbers of associated genes in the various comparisons (**B**). There were no consistent differences in the observed and expected numbers of genes associated to estrogenic or non-estrogenic DMR in the sperm fractions sorted based on CMA3− uptake of healthy donors and infertile patients

**Fig. 6:**
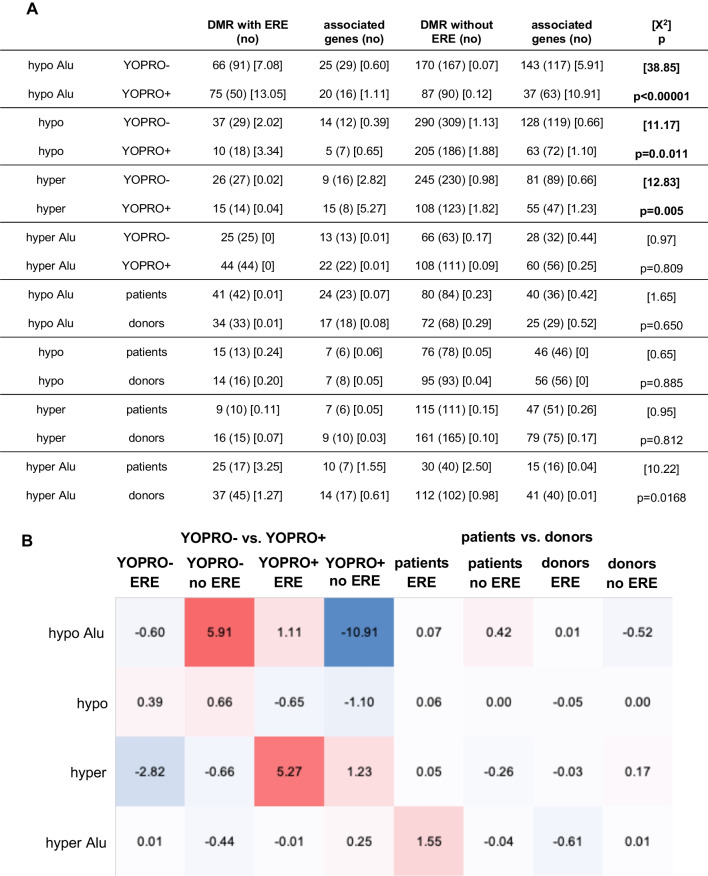
Genes associated to estrogenic or non-estrogenic transposable regulatory sequences in sperm fraction sorted based on YOPRO− uptake. The observed number of genes associated to the DMR in the sorted sperm fractions of infertile patients and healthy semen donors and their relationship with ERE in the regulatory segments of those genes were compared with the expected numbers and differences between both were evaluated using chi-squared (X^2^)-tests (**A**). Contrasting differences between the observed and the expected numbers of genes associated to hypomethylated non-estrogenic Alu sequences were detected in the YOPRO− sorted sperm fractions. In addition, more genes were detected associated with hypermethylated short transposable regulatory sequences in the YOPRO− positive sperm fractions (*p* = 0.005). Based on the X^2^-values, heat maps were constructed to visualize differences between observed and expected numbers of associated genes in the various comparisons (**B**)

We next examined the proportion of genes associated to the DMR in the various sorted sperm fractions of infertile patients and healthy semen donors, as given by the analytical step 9 in the Additional file 1: Tables S1 to Additional file 3: Table S3. Names of genes were recovered using the UCSC Genome Browser https://www.genome.ucsc.edu/ based on coordinates. We evaluated whether the proportion of associated genes was different in the sorted sperm fractions that contained DMR with ERE and compared to those without ERE (Figs. 5 and 6). We again compared the observed numbers with the expected numbers in 2 × 4-contingency tables and assessed the differences with the chi-squared test (DoF: 3). There were no consistent statistically significant differences between the observed and expected numbers of genes associated with estrogenic differentially methylated sequences in the CMA3 sorted sperm fractions (Fig. 5).

Fewer than expected genes were observed associated with hypomethylated non-estrogenic Alu sequences in the YOPRO+ sperm fractions, whereas more than expected genes were observed associated with hypomethylated non-estrogenic Alu sequences in the YOPRO− sperm fractions (*p* < 0.0001, Fig. 6). Furthermore, more genes were detected associated with hypermethylated short transposable regulatory sequences in the YOPRO− positive sperm fractions (*p* = 0.005).

### Pathways analyses

Pathways analyses were carried out online using STRING https://string-db.org/ and revealed that estrogenic hypomethylated Alu sequences in the YOPRO+ sperm fraction of infertile patients were associated with an enrichment of the histone acetyltransferase complex, with the SAGA-complex (Spt-Ada-Gcn5 acetyltransferase), with the PCAF-complex (P300/CBP-associated factor), and with the STAGA-pre-snoRNP-complex (GO:0000123, GO:0000124, GO:0000125, GO:0030914, GO:0033276, GO:0070761, respectively) due to the association with the AK6 and TAF9 genes. In turn, estrogenic hypomethylated Alu sequences in the CMA3+ fraction of infertile patients were associated with an enrichment of genes involved in the internal male genital organs (BTO:0003096), the testis (BTO:0001363) and the male reproductive gland (BTO:0000080), including the CATSPERG gene.

## Discussion

We hypothesized that changes in DNA methylation in the genomes of spermatozoa from infertile men with shortened AGD would be more associated with TEs containing ERE than their fertile counterparts, as these men may have been exposed antenatally to endocrine disruptors. Among all short TEs, many Alu sequences have previously been demonstrated to contain ERE [48, 49]. During primordial germ cell development, in post-implantation embryonic development, global DNA demethylation may allow these ERE elements to become more accessible. These accessible EREs in the regulatory sequences of the genome render the proliferating germ cells more susceptible to the signaling activities of environmental estrogenic endocrine disruptors during gametogenesis. We expect that random integration of short TEs containing ERE into the demethylated genome of early primordial germ cells and exposure of the organism to environmental endocrine disruptors during gametogenesis lead to the formation of cohorts of spermatozoa with subtle differences in gene regulation.

Candidate infertile patients were selected based on shortened AGD. Shortened AGD results from a combination of anti-androgenic [50, 51] and estrogenic disrupting effects [52] and is an accepted anthropomorphic endpoint measure of masculinization in reproductive toxicology [53]. We hypothesized that the spermatozoa from these infertile men, suspected to have been exposed to endocrine disruptors during antenatal development, would be variable and analysis of total sperm may mask important differences involved in their fertility. Thus, we used FACS to isolate cohorts of spermatozoa with characteristics known to be involved in reduced male fertility: 1. DNA fragmentation and enhanced membrane permeability (as given by YOPRO− uptake [54]) and 2. enhanced histone retainment and protamine deficiency (as given by CMA3− uptake [55–58]). Both DNA fragmentation and protamine deficiency have been associated with chromatin decondensation, which correlates with male fertility [36, 59–62]. Fertile and healthy semen donors were asked to provide a semen sample for control purposes. Semen counts of the participating infertile men and the healthy semen donors were similar (Table 1), suggesting a more subtle form of sperm dysfunction may be responsible for the differences in reproductive outcomes.

Comparisons of the normalized CpG methylation levels in the spermatozoa of both participant groups were similar (Fig. 2A). When comparing DNA methylation between FACS-sorted sperm cohorts stained with CMA3, lower CpG methylation levels were detected in the CMA3− negative sperm fraction (*p* = 0.039, Fig. 2B, C), a fraction enriched in highly condensed, protamine-rich chromatin. In all groups, the variance of the normalized CpG methylation levels was considerable, in agreement with the concept of sperm cohorts with distinct properties.

Further characterization of the sperm cohorts was carried out based on classification of the sorted sperm fractions in both participant groups based on the DNA methylation status of short TEs in the genome, either with or without ERE (Figs. 3 and 4). In agreement with the initial hypothesis, the fraction of sorted spermatozoa with decondensed chromatin (CMA3+) of infertile patients was significantly more likely to contain estrogenic Alu sequences (*p* = 0.0137, Fig. 3), whereas in reverse, the fraction of sorted spermatozoa with condensed protamine-rich chromatin (CMA3−) of infertile patients was less likely to contain hypermethylated estrogenic Alu sequences (*p* = 0.003, Fig. 3). Hypermethylation of regulatory sequences may appertain to inactivation of potential disruptive genes and be a protective element [63, 64].

The fraction of sorted spermatozoa of infertile patients stained with YOPRO (YOPRO+) was more likely to contain hypomethylated estrogenic Alu sequences (*p* < 0.001, Fig. 4), which corresponds to our initial hypothesis that abnormal, apoptotic spermatozoa are more likely to contain non-methylated estrogenic short transposable sequences regulating gene function. In reverse, the YOPRO− sperm fraction of infertile patients was more likely to contain estrogenic hypermethylated Alu sequences (*p* = 0.004, Fig. 4), which may be interpretated as the result of a protective element [63, 64]. Furthermore, the fraction of YOPRO− spermatozoa of infertile patients was more likely and the fraction of YOPRO+ spermatozoa of healthy donors was less likely to contain estrogenic short transposable sequences (*p* = 0.007, Fig. 4). These observations suggest a preponderance of estrogenic hypomethylated Alu sequences within abnormal sperm fractions of infertile patients, as measured by high levels of CMA3 or YOPRO. Aberrant methylation patterns in the genome of ejaculated spermatozoa [65] and in trophectoderm biopsies of blastocyst embryos generated via assisted reproduction with spermatozoa from infertile men with poor quality semen [66] have been implicated in the etiology of male infertility. Only few genome-wide analyses of the epigenome of human sperm have been carried out so far. One study focused on genome-wide DNA changes induced by physical exercise in 24 men [67]. Two other studies studied the influence of the folate metabolism on the epigenome of human spermatozoa in 30 men [68, 69]. Another study measured age-related changes in the epigenome of spermatozoa of 94 participants [70].

In this study, the infertile men were significantly older than their fertile counterparts (*p* = 0.006, Table 1). Paternal age is known to have an impact on the methylation profile of their spermatozoa, whereas age-dependent changes were typically observed in the sub-telometric regions of the genome, and there is no evidence that advanced paternal age modifies those regions of the genome that contain ERE [71].

The present study is the first to examine genome-wide changes in the DNA methylation patterns of sperm subpopulations FACS-sorted based on specific features, such as chromatin density and apoptosis. We provide evidence for a higher proportion of estrogenic transposable regulatory sequences in protamine-deficient or apoptotic sperm cohorts of infertile men. Activation of ERE implies the presence of functional genomic estrogen-mediated signaling pathways in the reproductive tissues during gametogenesis [72]. Both the estrogen receptors-alpha and -beta have been shown to be expressed in male reproductive tract. Knockout of the estrogen receptor alpha-gene results in impaired male fertility in mice [73]. However, treatment of male adult rats with agonists and antagonists of the estrogen receptor-beta causes DNA methylation defects, thereby reducing fertility outcome through enhanced resorption of post-implantation embryos [74, 75]. This effect was attributed to down-regulation of testicular DNA-(cytosine-5)-methyltransferase enzymes (DNMT) by estrogens. The human genome encodes five DNMT enzymes: DNMT1, DNMT2, DNMT3a, DNMT3b and DNMT3L [76]. DNMTs are centrally involved in both maintenance methylation and re-methylation during early germ cell development and later spermatogenesis. Co-localization and immunoprecipitation studies have demonstrated that estrogen receptor-beta, DNMT1 and the proliferating cell nuclear antigen (PCNA, a DNA replication nuclear transcription factor) jointly interact at the level of ERE’s [77]. Particularly DNMT1 and DNMT3L have been shown to be involved in silencing parasitic sequence elements including TEs during gametogenesis [22, 78, 79].

The more observed than expected numbers of estrogenic hypermethylated Alu sequences in the sperm fractions of healthy donors remaining unstained by CMA3 and in sperm fractions of infertile patients remaining unstained by YOPRO may be indicative of a protective effect of hypermethylation in estrogenic TEs. Re-methylation of TEs potentially regulating disruptive genes during germ cell formation has been suggested to be essential for maintenance of future fertility [63, 64].

We next sought to determine whether DMRs from ERE versus non-ERE transposable elements showed different associations with neighboring genes (Figs. 5 and 6). We found no consistent difference between the number of genes near ERE-containing versus non-ERE-containing TE’s in sperm sorted by CMA3 levels (Fig. 5). In contrast, whereas more genes were associated to non-estrogenic hypomethylated Alu sequences in YOPRO− sperm fractions of infertile patients, fewer genes were associated to the same non-estrogenic hypomethylated Alu sequences in YOPRO+ sperm fractions (*p* < 0.00001, Fig. 6). Lists of the genes associated to estrogenic or non-estrogenic regulatory sequences are given in Additional file 4: Table S4 (CMA3) and in Additional file 5: Table S5 (YOPRO). Among the genes associated with differentially methylated regions are CATSPERG, which is associated to an estrogenic hypomethylated Alu sequence, more likely to be present in the CMA3+ sperm fraction of infertile patients, and CATSPERZ, which is associated to an estrogenic hypomethylated Alu sequence more likely to be present in the CMA3− sperm fraction of infertile patients. CATSPERG and CATSPERZ are auxiliary proteins of the CATSPER calcium-selective ion channel complex, which is testis- and sperm-specific and essential for hyperactivated motility of capacitated spermatozoa [80]. Pathway analysis of genes associated to estrogenic hypomethylated Alu sequences in the CMA3+ fraction of infertile patients demonstrated an enrichment of genes involved in the formation and function of internal male genital organs.

These associations demonstrate that cohorts of spermatozoa of infertile men with shortened AGD, as a clinical marker of antenatal exposure to anti-androgenic or estrogenic endocrine disruptors, carry differentially methylated regions involved in regulating sperm function, such as chromatin density and apoptosis. Comparison of the number of differentially methylated TEs in infertile patients versus those of healthy semen donors demonstrates a preponderance of estrogenic hypomethylated Alu sequences in protamine-deficient and in apoptotic sperm fractions in the former, that may contribute to their reduced fertility. In addition, more hypermethylated estrogenic Alu sequences were detected in the protamine-rich sperm fractions of fertile semen donors (*p* = 0.003, Fig. 3) and in the non-apoptotic sperm fraction of infertile patients (*p* = 0.004, Fig. 4), which we postulate as protective elements.

We are aware that various aspects of the study protocol bare limitations, such as the few participants included, the in part unproven association between the shortened AGD with antenatal exposure to endocrine disruptors, the limited diagnostic accuracy of RRBS (e.g., 70–80% [81]) and the focus on ERE only in the regulatory sequences of the genome. However, for the first time, it was now possible to demonstrate a link between the increased presence of hormonal response elements in differentially methylated regulatory sequences of the genome of sorted sperm fractions of infertile men suspected to have been exposed to endocrine disruptors based on a well-established anthropomorphic parameter in reproductive toxicology. This or similar mechanisms may well be responsible for the impairment of male fertility, the higher incidence of congenital malformations, such as hypospadias and cryptorchidism, and the higher incidence of testicular cancer in young adults in many industrialized countries. However, the current study was carried out in men with normal conventional semen parameters and idiopathic infertility, and future studies may focus on men with more pronounced impairment of spermatogenesis. The mechanisms presented here may be elaborated further to confirm the existence of the hitherto hypothetical testicular dysgenesis syndrome [10, 82, 83].

## Methods

### Recruitment of infertile patients and fertile donors

For this experimental study, eleven infertile men and ten fertile semen donors were recruited in the Institute of Reproductive Medicine and Gynecological Endocrinology (RME) at the University Hospital of the University of Basel, Switzerland. The study was approved by the local ethical committee (EKNZ 2017-01407). All invited participants were informed about the rationale of the study and signed the consent form. They were asked to produce a semen sample after a recommended abstinence of two to seven days, according to the WHO-guidelines (2010). None of the participants underwent any specific treatment.

The criteria for recruitment of the eleven infertile participants consisted of duration of infertility of at least 12 months, normal semen quality, as given by conventional semen analysis, AGD of 40 mm or less [84] and > 20% fragmentation of DNA in the nuclei of swim-up spermatozoa, as given by terminal deoxynucleotide transferase-mediated dUTP nick-end (TUNEL) labeling [85]. The control group consisted of 10 healthy men, who previously had donated normal semen for donor insemination. In addition to personal history, all participants underwent physical examination including measurement of their testicular volumes and of AGD. The serum levels of LH, FSH and total testosterone were measured as well.

### Initial processing of the semen sample

Semen samples were collected through masturbation. After collection, the semen samples were allowed to liquify for 30 min at 37 °C and then processed for semen analysis following the WHO-guidelines (2010). The fraction to be isolated for FACS was washed once with modified Ham’s F-10 (MHF-10 media) and then re-suspended in MHF-10 media at a concentration of 10 million sperm per ml.

### YO-PRO-1-staining

Spermatozoa labeled with the YO-PRO-1 dye are undergoing or have undergone apoptosis, as the YO-PRO-1-dye readily enters cells through defects in the membrane (Fig. 1). YO-PRO-1-negative spermatozoa are characterized by low DNA fragmentation, as demonstrated by TUNEL [54]. Initially, the spermatozoa were stained with 0.2 µM of the YO-PRO-1 dye (Thermo Fisher Scientific) and with 1 µg/ml of the Hoechst 33342 dye to exclude debris, then diluted in MHF-10 media for 30 min at room temperature in the dark. For the validation, spermatozoa were also stained with 0.2 µM of the YO-PRO-1 dye and 5 µM of the Vybrant Dye Cycle Ruby (Thermo Fisher Scientific) for 30 min at room temperature in the dark. After staining, spermatozoa were immediately sorted using a BD Influx cell sorter. YO-PRO-1-positive sorted sperm populations were labeled as YOPRO+, whereas YO-PRO-1-negative sorted sperm populations were labeled as YOPRO−.

### Chromomycin A3 (CMA3)-staining

Staining with the CMA3− dye is widely used for indirect assessment of protamine deficiency in a semen sample [55–58]. CMA3 is a fluorochrome specific for guanosine cytosine-rich sequences in the genome that compete with protamine in binding to DNA (Fig. 1). CMA3− positive spermatozoa are enriched in histone, whereas sperms enriched in protamine remain CMA3− negative. Spermatozoa were centrifuged at 500 × g for 10 min at room temperature and re-suspended at 5 million sperm per ml in McIlvaine’s buffer, pH 7.0 + 10 mM MgCl_2_. For initial rounds, sperms were stained with 0.25 mg/ml CMA3 (Sigma-Aldrich) and with 1 µg/ml Hoechst 33342 diluted in McIlvaine’s buffer, pH 7.0 + 10 mM MgCl_2_ for 30 min in the dark. For the validation round, sperms were stained with 0.25 mg/ml CMA3 dye and 5 µM Vybrant Dye Cycle Ruby (Thermo Fisher Scientific) for 30 min in the dark at room temperature. After staining, sperms were immediately sorted using a BD Influx cell sorter. CMA3− positive sorted sperm populations were labeled as CMA3+, whereas CMA3− negative sorted sperm populations were labeled as CMA3−.

### Separation of spermatozoa using FACS

Sorting of spermatozoa was carried out with a BD Influx cell sorter equipped with three lasers (355 nm, 488 nm and 640 nm) after swim-up preparation, as described earlier [54]. The FACS sorting device, equipped with an air purification hood, was installed next to the spermatology laboratory and was exclusively used for the sorting of human spermatozoa. Gating strategy involved first removal of debris by excluding events with very low forward scatter (FSC). Following this, spermatozoa were selected based on DNA content (Hoechst 33342 or Vybrant Dye Cycle Ruby). Finally, selected spermatozoa were separated based on the level of the desired dyes (YO-PRO-1 or CMA3) and collected into 15 ml Falcon tubes (Fig. 1). Cells used for RRBS were centrifuged at 500 × g for 5 min at room temperature and flash frozen in pellets that were stored at − 80 °C until further processing. To validate the quality of sorting, 10–15,000 sperms from each sorted population were placed onto a 10-well diagnostic slide, mounted with Vecta Shield mounting media (Vector labs) and imaged by fluorescence microscopy for the dyes used in sorting.

### DNA extraction from sorted populations of spermatozoa

DNA was extracted from FACS-sorted spermatozoa as previously described [86]. Lysis was performed overnight at 55 °C with constant agitation at 150 t/min in 300 µl lysis solution from the Puregene Gentra kit (Qiagen, Cat No. 158745). The lysis solution was supplemented with three units of proteinase K (5 µl of 20 µg/µl solution from Fermentas at 600 U/ml, catalog No. EO0491) and 1.5 µl of 1 M DL-Dithiothreitol (DTT, Applichem, catalog no. A1101.0005). After removal of the protein fraction, DNA was precipitated in 100% isopropanol, washed with 70% ethanol, and re-suspended in 100 µl of water. We quantified intact double-strand DNA (dsDNA) on a Qubit™ Fluorometer by incorporation of the Pico Green dye (Life Technologies, USA) and DNA quantities with the Nano Drop® ND-1000 (Thermo Scientific).

### DNA fragmentation analysis using capillary electrophoresis

DNA fragmentation was analyzed by capillary electrophoresis using a genomic DNA tape station system (Agilent, 2200 Tape Station Nucleic Acid system, G2965AA). 1 µl of DNA per sample was loaded on a Genomic DNA D5000 Screen Tape (5067-5592) using the high sensitivity D5000 reagents (5067-5593). Data were processed using the Agilent Software packages (2200 Tape Station Controller Software and Tape Station Analysis software). The DNA integrity number (DIN) was used to determine the fragmentation of a genomic DNA sample.

### RRBS libraries and sequencing

DNA cytosine methylation was analyzed on a genome-wide scale using reduced representation bisulfite sequencing (RRBS, Diagenode®, Cat. G02020000). RRBS libraries were prepared using the Diagenode® Premium RRBS technology [87]. Briefly, *MspI* cuts CCGG sites in DNA samples at 37 °C for 12 h before ends repair. Diagenode® ligates the adaptors with samples related specific barcodes. Bisulfite treatment on libraries converts un-methylated cytosine to uracil before PCR amplification of the libraries. Methylated cytosine remains unchanged. The quality of the final libraries was checked on an Agilent 2100 High Sensitivity DNA chip to identify the signature of Human RRBS libraries characterized by the presence of pics at 200, at 260 and at 330 base pairs (bp). The concentration was determined by performing qPCR on the samples using a dilution of PhiX index3 as standard. Libraries were sequenced in paired-end mode of 50 bp in a HiSeq4000 instrument (Illumina Inc.) using 1 full-lane for 5 multiplexed samples.

### RRBS datasets

The amount of DNA of 76 out of 84 (90.5%) of the FACS-sorted sperm samples was sufficient to produce sequenced and analyzable RRBS libraries. Each sequenced RRBS library is associated with a unique identification number (HS11 to HS104, see Additional file 1: Tables S1, Additional file 2: Table S2 and Additional file 3: Table S3). As in some sorted samples insufficient DNA was extracted for analysis, RRBS data correspond to the CMA3− negative (CMA3−) sperm fractions collected in 10 healthy semen donors and nine infertile patients, the CMA3− positive fraction (CMA3+) collected in nine donors and nine patients, the YOPRO− sperm fraction collected in nine donors and nine patients and the YOPRO+ fractions collected in 10 donor and 11 patients. Globally, sufficient DNA of all 4 FACS-sorted sperm fractions (YOPRO+, YOPRO−, CMA3+ and CMA3−) was successfully obtained in eight donors (32 samples) and seven patients (28 samples). In addition, we obtained RRBS data on three fractions only of two (six samples), one donor lacking sufficient DNA of the CMA3+ and another donor lacking sufficient DNA of the YOPRO− sorted fractions. Finally, in one patient, two CMA3− fractions were produced, but the YOPRO− negative sample was missing, in two patients, both CMA3+ and CMA3− fractions were missing (four samples) and in one patient only the CMA3+ and YOPRO+ fractions were obtained (two samples).

### Methylation extraction

Analyses of RRBS data were performed from the fastq files using a bioinformatics pipeline, written in Bash, Python and R. Matthias Beyens (BISC Global, Ghent, Belgium) developed this pipeline directly in the high performance computing cluster (HPC) belonging to the University of Geneva, as previously reported [88]. Briefly, the pipeline performed first reads trimming using Trim Galore (version 0.6.0) and cut-adapted (version 2.3). Then, the reads were mapped against the human bisulfite genome (assembly GRCH38) and methylation levels extracted using Bismark (version 0.22.1) running with Bowtie 2 (version 2.3.5.1). We used the 3-letter aligner Bismark to avoid overestimation of methylation level [89]. To avoid methylation bias, we discarded the methylation levels coming from the three first bases located at both ends (5’ and 3’) of reads 2 and at the 5’ extremity of read1. Finally, we filtered out the methylation values located in a C/T single nucleotide polymorphism (SNP) in the Bismark coverage files (*.bismark.cov.gz), due to the impossibility to distinguish such SNP from methylation variations (a mean of 210′087 SNP per sample were filtered in the dataset). To ensure that the same analytical process was applied to each sample, we processed all fastq files in parallel in the HPC. We recorded in a log sheet the entire process starting with the fastq files and ending with the filtered methylation values. Note that, both efficiencies of *MspI* digestion and bisulfite conversion were estimated based on the obtained sequences. The proportion of GG in read1 at position 2 and 3 resulted in a median at 96% for *MspI* digestion efficiency. We estimated the bisulfite conversion efficiency based on 100 minus the methylation outside CpG (CHG context) resulting in 99.5%, as well as based on the base repair process by reporting the proportion of A in reads 2 at position 2, resulting in a median of 95%. We reported the global CpG methylation levels from the Bismark reports. We removed batch effects in these values using the scale function in R for each batch and by producing the standardized mean CpG methylation levels. Note that, the three batches previously mentioned did not differ in their composition, in terms of donors and patients, as well as in FACS types.

### Pairwise comparisons for differential methylation

As summarized in the Additional file 1: Tables S1, Additional file 2: Table S2 and Additional file 3: Table S3, we conducted nine pairwise comparisons (analytical step 1, “Samples and contrast”) for differential methylation using the BSmooth package [90]. We recovered both differentially methylated regions DMR and differentially methylated CpGs and used two different analytical methods to identify DMR in the matched samples. First, the “Fisher tests”-function in the Bsseq package was used to identify differential methylation at each CpG site individually (analytical step 2, “CpG”), based on 2 by 2 contingency tables. We first established the significance threshold (analytical step 3, “adjusted p”) before retaining CpG’s with Fisher tests-derived p values resisting Bonferroni correction for multiple testing (analytical step 4, “CpG adjusted”), and with a finite value for the logarithm in base 2 of the odds ratio (analytical step 5, “Remove INF”). Infinite values of log2(OR) resulting from division by zero were not considered for further analysis. Second, we identified DMR using the smoothing algorithm BSmooth R (analytical step 6, “DMR”). The quantile-based cutoff of the t-statistic was set at 0.025 and 0.975, respectively, and methylation changes had to be present in at least three samples within each patient group with an absolute change in methylation of ≥ 0.25. Next, we validated each DMR (analytical step 7, “overlapping DMR”) and the CpG (analytical step 8, “overlapped CpG”) by their overlaps using Genomic Ranges-derived functions. This double analytical verification process was carried out to obtain concordant evidence. DMRs were annotated depending on their overlap with genes (analytical step 9, “Genes”) reporting the official gene symbols, as well as with the following genes structures: “1to5kb” (5 kb region upstream of promotors), “promotors,” “5UTRs,” “3UTR,” “exons,” “introns,” “intronexonboundaries” (200 bp up/down stream of any boundary between an exon and intron). CpGs were annotated depending on their distance to a CpG island (CGI) as “islands” (overlapping), “shores” (2 kb of distance form on end), “shelf” (> 2 kb < 4 kb distance), and “inter” (> 4 kb) using the Bioconductor package “annotatr” [91]. As the mode of the frequency distributions of the normalized CpG methylation levels in the various groupings varied from one analysis to another, the statistical significance of the differences was examined both with ANOVA and with Kruskal–Wallis (KW).

### Analysis of associated genes and pathways

Genes associated to estrogenic or non-estrogenic hypo- or hypermethylated regulatory sequences were recovered using the UCSC Genome Browser www.genome.ucsc.edu based on the DMR coordinates. In each pairwise comparison, we submitted online the recorded gene names in STRING [92]. We recovered the number of affected biological pathways (analytical step 10, “Pathways”) and grouped these pathways according to those involved in the localization of the encoded protein, those acting on DNA, on RNA, on protein as well as those occurring at the cellular level.

### Identification of differential methylation within Alu repeat sequences

We extracted the coordinates of 1′262′425 Alu repeats from the human genome by using the UCSC Table Browser (http://genome-euro.ucsc.edu/cgi-bin/hgTables). The following options were installed online (Genome options: clade = Mammal, genome = Human, assembly = GRCh38/hg38; group = Repeats, track = RepeatMasker, table = rmsk, region = genome, Filter edited with “repFamily” that does match “Alu,” output format = BED). The file created (Alu.txt) contained the coordinates of all known Alu repeats present in the haploid human reference genome (3.1 Giga base) and representing ~ 15% of the sequence length (0.45 Giga bases). We retained 1′238′897 Alu sequences concordant with the bioconductor package “BSgenome.Hsapiens.UCSC.hg38” and discarded 23′528 discordant Alu sequences. Fifty-one different Alu subfamilies were detected, among which nine subfamilies were enriched in monomers (Alu, AluYa8, AluYh9, AluYk11, AluYk12, FAM, FLAM_A, FLAM_C and FRAM) and 42 were dimeric (AluJb, AluJo, AluJr, etc.). We systematically screened the differentially methylated CpG (analytical steps 7) and DMR (analytical step 8) for their overlaps within these concordant Alu repeats by using “Genomic Ranges” derived functions.

### Enrichment analysis for differentially methylated Alu repeats with ERE

First, we uploaded the HOmo sapiens COmprehensive MOdel COllection (HOCOMOCO) derived probability weight scoring matrix referred as “ESR1_HUMAN.H11MO.1.A” (https://hocomoco11.autosome.ru/motif/ESR1_HUMAN.H11MO.1.A) in order to identify estrogen response elements (ERE) specific for the estrogen receptor-1 (ESR1). We used the threshold for genome-wide significance set at 7.6. That probability weight matrix for ESR1 binding sites was passed into a single sequence containing the 1′238′897 concordant Alu that were concatenated. Each of the Alu sequences containing at least one positive ESR1 binding site with a score higher than 7.6 was considered as a putative estrogenic Alu sequence (180′789), whereas the others were considered as non-estrogenic (1′058′101). 17.1% of all Alu sequences tested contained ERE and were putatively considered as estrogenic. Then, we used the same analytical approach on the CpG, segregating the hypomethylated from the hypermethylated CpG across all pairwise comparisons to identify those that are overlapping non-estrogenic and estrogenic Alu. Differences in the observed number of ERE-containing Alu sequences and other short transposable sequences from the expected numbers in the various sorted sperm populations were analyzed using Chi-squared analysis.

### Analysis of imprinted genes

Imprinting refers to the process implying DNA methylation mediated silencing of gene resulting in a copy of the gene turned off in a parent-of-origin-dependent manner [93]. We aimed at estimating the differential methylation across the tested sperm subtypes in imprinted genes. First, 274 imprinted genes (11 unknown, 2 random, 127 paternal, 107 maternal, 4 isoform-dependent, 18 biallelic and 5 not assigned) were recovered from the Gene imprint portal (https://www.geneimprint.org, downloaded in October 2021). We started the analysis by selecting the DMR detected across the comparison by the Bsseq-algorithm and containing the imprinted genes names in its annotation symbol. From the selected DMR, we recovered the overlapping CpG that showed differential distribution of methylation and un-methylation calls according to the Fisher tests. We then extracted all methylation and un-methylation calls and methylation levels across all samples in these sites. Wilcox tests were then performed for assessing differential methylation levels across groups considering the same sperm subtypes and comparing donors to patients (for example: CMA3+ donor vs. CMA3+ patient) or comparing the complementary sperm subtypes in either donors or patients (for example: CMA3− donor vs. CMA3+ donor).

## Supplementary Information

**Additional file 1. Table S1**: Comparison of the cumulated epigenomic data of the washed spermatozoa of semen donors with the cumulated epigenomic data of the washed spermatozoa of infertile patients, followed by stepwise analysis of the results.

**Additional file 2. Table S2**: Pairwise comparison of the epigenomic data according to the staining and sorting results.

**Additional file 3. Table S3**: Pairwise comparison of the epigenomic data between donors and patients, ranked according to the staining and sorting results.

**Additional file 4:** Genes associated to regulatory TE in sperm fractions sorted based on CMA3.

**Additional file 5:** Genes associated to regulatory TE in sperm fractions sorted based on YOPRO.

## Data Availability

RRBS data are available from the Gene Expression Omnibus of the National Center for Biotechnology Information under the accession number GSE199920.
