# Supplementary material for: Altered DNA methylation in estrogen-responsive repetitive sequences of spermatozoa of infertile men with shortened anogenital distance
Source: Clin Epigenetics. 2022 Dec 26;14:185. doi: 10.1186/s13148-022-01409-1 (PMC9793642; doi:10.1186/s13148-022-01409-1)
Supplement: Supplementary file 1 — Additional file 1. Table S1: Comparison of the cumulated epigenomic data of the washed spermatozoa of semen donors with the cumulated epigenomic data of the washed spermatozoa of infertile patients, followed by stepwise analysis of the results. [file 13148_2022_1409_MOESM1_ESM.docx]

**Supplementary Table 1:**

Comparison of the cumulated epigenomic data of the washed spermatozoa of semen donors with the cumulated epigenomic data of the washed spermatozoa of infertile patients, followed by stepwise analysis of the results.

| analysis pathway | Analysis 1: donor vs. patient, cumulated |
| --- | --- |
| 1. samples and contrast | **38 sorted sperm samples of semen donors**  [HS11, HS12, HS13, HS14, HS15, HS16, HS17, HS18, HS19, HS20, HS21, HS22, HS31, HS32, HS33, HS62, HS63, HS64, HS69, HS70, HS75, HS76, HS77, HS78, HS81, HS82, HS83, HS84, HS89, HS90, HS95, HS96, HS97, HS98, HS101, HS102, HS103, HS104]  **vs.**  **38 sorted sperm samples of infertile patients**  [HS23, HS24, HS25, HS26, HS27, HS28, HS29, HS30, HS35, HS36, HS37, HS38, HS40, HS43, HS53, HS54, HS58, HS59, HS65, HS66, HS67, HS68, HS71, HS72, HS73, HS74, HS79, HS80, HS85, HS86, HS87, HS88, HS91, HS92, HS93, HS94, HS99, HS100] |
| 2. CpG | 6’955’288 |
| 3. adjusted p | 1.4E-09 |
| 4. CpG adjusted | 471’703 |
| 5. remove INF | 301’749 |
| 6. DMR | 102 |
| 7. overlapping DMR | 8 |
| 8. overlapped CpG | 32 |
| 9. genes | 7 |
| 10. pathways | 2 |
