# Supplementary material for: Altered DNA methylation in estrogen-responsive repetitive sequences of spermatozoa of infertile men with shortened anogenital distance
Source: Clin Epigenetics. 2022 Dec 26;14:185. doi: 10.1186/s13148-022-01409-1 (PMC9793642; doi:10.1186/s13148-022-01409-1)
Supplement: Supplementary file 2 — Additional file 2. Table S2: Pairwise comparison of the epigenomic data according to the staining and sorting results. [file 13148_2022_1409_MOESM2_ESM.docx]

**Supplementary Table 2:**

Pairwise comparison of the epigenomic data according to the staining and sorting results.

| analysis pathway | Analysis 4: patients CMA3- vs. patients CMA3+ | Analysis 5: donors CMA3- vs. donors CMA3+ | Analysis 6: donors YOPRO- vs. donors YOPRO+ | Analysis 7: patients YOPRO- vs. patients YOPRO+ |
| --- | --- | --- | --- | --- |
| 1. samples and contrast | **9 patients CMA3-** [HS26, HS28, HS36, HS37, HS85, HS87, HS91, HS93, HS99]  **vs.**  **9 patients CMA3+** [HS25, HS29, HS35, HS40, HS86, HS88, HS92, HS94, HS100] | **10 donors CMA3-** [HS11, HS15, HS20, HS32, HS62, HS89, HS95, HS97, HS101, HS103]  **vs.**  **9 donors CMA3+** [HS13, HS16, HS22, HS31, HS90, HS96, HS98, HS102, HS104] | **9 donors YOPRO-** [HS12, HS17, HS21, HS64, HS69, HS75, HS77, HS81, HS83]  **vs.**  **10 donors YOPRO+** [HS14, HS18, HS19, HS33, HS63, HS70, HS76, HS78, HS82, HS84] | **9 patients YOPRO-** [HS24, HS27, HS54, HS59, HS65, HS67, HS71, HS73, HS79]  **vs.**  **11 patients YOPRO+** [HS23, HS30, HS38, HS43, HS53, HS58, HS66, HS68, HS72, HS74, HS80] |
| 2. CpG | 3’605’530 | 4’238’993 | 4’527’835 | 4’879’054 |
| 3. adjusted p | 2.8E-09 | 2.4E-09 | 2.2E-09 | 2.0E-09 |
| 4. CpG adjusted | 173’875 | 175’026 | 176’236 | 106’483 |
| 5. remove INF | 69’367 | 89’345 | 77’817 | 54’335 |
| 6. DMR | 6’372 | 3’935 | 2’655 | 3’354 |
| 7. overlapping DMR | 854 | 552 | 314 | 260 |
| 8. overlapped CpG | 1’464 | 1’082 | 568 | 410 |
| 9. genes | 579 | 379 | 213 | 177 |
| 10. pathways | 6 | 11 | 52 | 7 |
