# Supplementary material for: Altered DNA methylation in estrogen-responsive repetitive sequences of spermatozoa of infertile men with shortened anogenital distance
Source: Clin Epigenetics. 2022 Dec 26;14:185. doi: 10.1186/s13148-022-01409-1 (PMC9793642; doi:10.1186/s13148-022-01409-1)
Supplement: Supplementary file 3 — Additional file 3. Table S3: Pairwise comparison of the epigenomic data between donors and patients, ranked according to the staining and sorting results. [file 13148_2022_1409_MOESM3_ESM.docx]

**Supplementary Table 3:**

Pairwise comparison of the epigenomic data between donors and patients, ranked according to the staining and sorting results.

| analysis pathway | Analysis 2: patient CMA3- vs.  donor CMA3- | Analysis 9: patient CMA3+ vs.  donor CMA3+ | Analysis 8: patient YOPRO+ vs. donor YOPRO+ | Analysis 3: donor YOPRO- vs. patient YOPRO- |
| --- | --- | --- | --- | --- |
| 1. samples and contrast | **9 patients CMA3-**  [HS26, HS28, HS36, HS37, HS85, HS87, HS91, HS93, HS99]  **vs.**  **10 donors CMA3-**  [HS11, HS15, HS20, HS32, HS62, HS89, HS95, HS97, HS101, HS103] | **9 patients CMA3+**  [HS25, HS29, HS35, HS40, HS86, HS88, HS92, HS94, HS100]  **vs.**  **9 donors CMA3+**  [HS13, HS16, HS22, HS31, HS90, HS96, HS98, HS104] | **11 patients YORPO+**  [HS23, HS30, HS38, HS43, HS53, HS58, HS66, HS68, HS72, HS74, HS80]  **vs.**  **10 donors YORPO+**  [HS14, HS18, HS19, HS33, HS63, HS70, HS76, HS78, HS82, HS84] | **9 donors YORPO-**  [HS12, HS17, HS21, HS64, HS69, HS75, HS77, HS81, HS83]  **vs.**  **9 patients YOPRO-**  [HS24, HS27, HS54, HS59, HS65, HS67, HS71, HS73, HS79] |
| 2. CpG | 3’702’338 | 3’947’187 | 4’928’623 | 4’455’216 |
| 3. adjusted p | 2.7E-09 | 2.5E-09 | 2.0E-09 | 2.2E-09 |
| 4. CpG adjusted | 173’651 | 182’142 | 173’323 | 131’022 |
| 5. remove INF | 81’458 | 79’870 | 86’344 | 61’470 |
| 6. DMR | 5’101 | 5’337 | 3’181 | 3’691 |
| 7. overlapping DMR | 836 | 763 | 360 | 469 |
| 8. overlapped CpG | 1’521 | 1’396 | 686 | 971 |
| 9. genes | 579 | 521 | 221 | 322 |
| 10. pathways | 14 | 78 | 0 | 4 |
